# LasR-regulated proteases in acute vs. chronic lung infection: a double-edged sword

**DOI:** 10.15698/mic2021.07.755

**Published:** 2021-05-31

**Authors:** Lisa C. Hennemann, Dao Nguyen

**Affiliations:** 1 Department of Microbiology and Immunology, McGill University, Montreal, Quebec, Canada.; 2 Meakins Christie laboratories, Research Institute of the McGill University Health Centre, Montreal, Quebec, Canada.; 3 Department of Medicine, McGill University, Montreal, Quebec, Canada.

**Keywords:** Pseudomonas aeruginosa, cystic fibrosis, airway epithelium, quorum sensing, LasR, neutrophilic inflammation, secreted proteases, host pathoadaptation

## Abstract

*Pseudomonas aeruginosa* is a gram-negative opportunistic pathogen capable of causing both acute and chronic infections, particularly in individuals with compromised host defenses. The quorum sensing transcriptional activator LasR is widely recognized for its role in regulating the expression of acute virulence factors, notably several secreted proteases which cause direct host damage and subvert host immunity in acute infections. Paradoxically, lung infections caused by LasR-deficient variants, which are found in at least a third of cystic fibrosis (CF) patients with chronic *P. aeruginosa* infections, are associated with accelerated lung disease and increased markers of inflammation compared to infections caused by strains with a functional LasR system. While the loss of LasR function often (although not always) results in impaired production of LasR-controlled acute virulence factors, the implication of this pathoadaptation on host-pathogen interactions and chronic disease pathology is less well recognized. We recently observed that loss of LasR function in *lasR* variants, which results in impaired secreted protease production, led to increased expression of the membrane-bound surface adhesion molecule mICAM-1 in the airway epithelium, and increased neutrophilic inflammation. Specifically, human airway epithelial cells stimulated with *lasR* variants had higher mICAM-1 expression and greater neutrophil binding *in vitro* compared to stimulation with wild-type *P. aeruginosa*. In a subacute non-lethal *P. aeruginosa* lung infection model, *lasR* variant infection also induced higher mICAM-1 expression in the murine airway epithelium and was associated with increased neutrophilic pulmonary inflammation *in vivo*. Here, we discuss how (loss of) LasR function and LasR-regulated proteases affect host immunity, inflammation and tissue pathology in acute vs. chronic *P. aeruginosa* lung infection.

*P. aeruginosa* can cause acute pneumonia, which is tissue invasive, disseminates systemically and is associated with a high mortality. In individuals with the genetic disease CF, *P. aeruginosa* causes chronic and often lifelong airway infections. While these chronic infections typically do not invade surrounding tissues nor disseminate, they fuel an excessive and unresolving neutrophilic inflammation, which releases abundant neutrophil elastase and reactive oxygen species, leading to tissue damage and progressive organ decline. How *P. aeruginosa* interacts with host cells and causes pathology thus differs in acute chronic infections, even when both involve the lung.

LasR directly or indirectly regulates the expression of up to 5% of the *P. aeruginosa* genome, including many genes that encode acute virulence factors such as the phenazine pyocyanin and several secreted proteases including LasA, LasB, AprA and type IV protease (T4P). *P. aeruginosa* secreted proteases have been extensively studied for their ability to cause host tissue damage and subvert host immunity. Numerous *in vitro* studies, including ours, indicate that LasR-regulated proteases, particularly LasB and AprA, can proteolytically degrade a broad range of host molecules (**Fig. 1**). LasR-regulated proteases can degrade cell junction and extracellular matrix proteins (e.g. occludin, VE-cadherin, elastin, collagen) and surface receptors (e.g uPAR, PAR-2) leading to the breakdown of cellular barriers and direct tissue damage. Inactivation of chemokines and immune mediators such as IL-8, IL-6 and ENA78 (CXCL5) can suppress host immunity and enable immune evasion, while proteolytic cleavage of surface proteins such as mICAM-1 can mitigate neutrophil transepithelial migration and adhesion, resulting in suppressed neutrophil responses to infection. Furthermore, degradation of IFN-γ suppresses anti-viral responses in airway epithelial cells, leading to increased viral spreading upon infection with human rhinovirus and respiratory syncytial virus.

**Figure 1:**
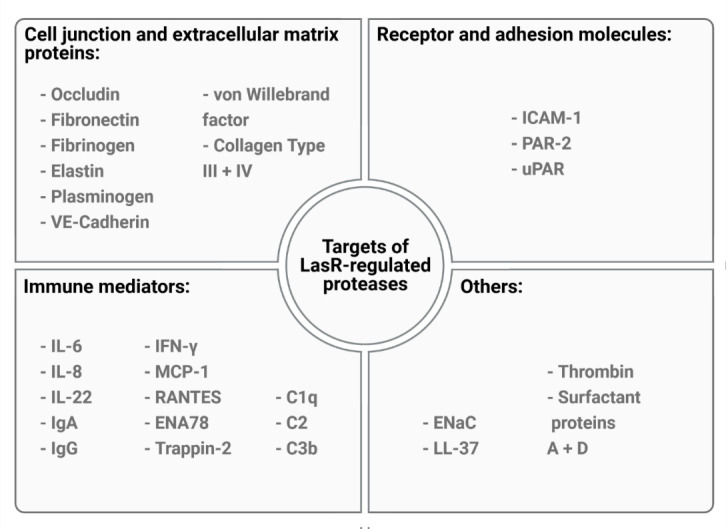
FIGURE 1: Targets of LasR-regulated proteases. LasR-regulated proteases can cleave a large number of host targets ranging from components of the extracellular matrix and tight junction to immune mediators, adhesion molecules and other molecules. Some targets, such as ICAM-1, can be degraded by multiple LasR-regulated proteases, while others such as ENaC are proteolytically activated rather than inactivated.

Since LasR-regulated proteases can be deleterious to the host in a multitude of ways, one would expect that loss of function *lasR* variants lacking LasR-regulated proteases, which are highly prevalent among clinical isolates from chronic CF infections, would cause less severe lung disease. Surprisingly, CF patients infected with *lasR* variants show accelerated decline in lung function and increased IL-8 levels, a marker of neutrophilic inflammation, compared to patients infected with wild-type *lasR* strains. Past and present studies in our lab have aimed at understanding the mechanisms that contribute to this apparent paradoxical outcome.

We propose that the interactions between LasR-regulated exoproducts and the host vary and have different implications to disease pathology and severity in acute vs. chronic infection settings (**Fig. 2**). In acute infections, *P. aeruginosa* is likely motile, expresses functional LasR and a large array of acute virulence factors, with LasR-regulated proteases causing direct tissue damage by degrading extracellular matrix proteins and cell junctions. While host epithelial cells express pro-inflammatory surface receptors and produce high levels of pro-inflammatory cytokines (e.g. IL-8, ENA78) upon stimulation by *P. aeruginosa*, LasR-regulated proteases can counteract these responses by directly degrading inflammatory and immune mediators. LasR-mediated interactions thus dampen immune responses, allowing for immune evasion, bacterial proliferation and direct tissue damage. These deleterious interactions likely dominate during acute infection settings and explain the attenuated virulence of the *lasR* mutant observed in various acute infection models.

**Figure 2:**
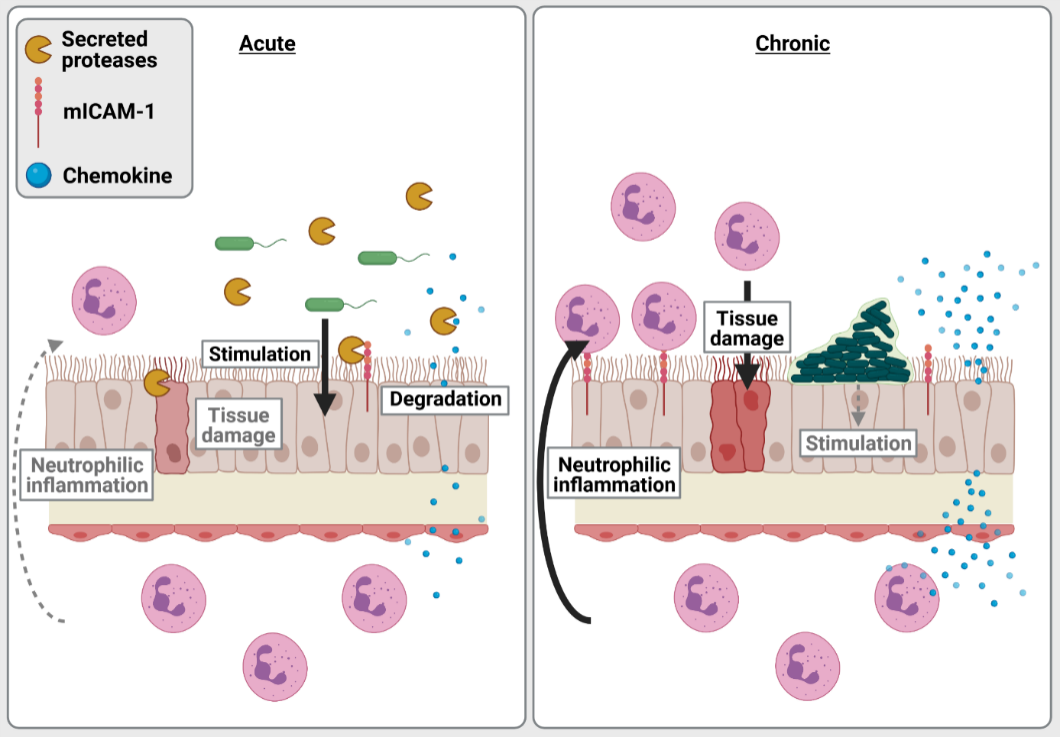
FIGURE 2: LasR-regulated proteases and neutrophilic inflammation in acute vs. chronic lung infection. In acute infection (left) such as in acute pneumonia, *P. aeruginosa* strains typically have a functional LasR system and express various extracellular virulence factors, leading to stimulation of the airway epithelium and immune cells to produce pro-inflammatory chemokines, cytokines and mediators such as mICAM-1. LasR-regulated proteases degrade pro-inflammatory mediators thus following *P. aeruginosa* to evade host immunity, dampen neutrophilic responses while causing direct damage to host cellular barriers and extracellular matrix. In chronic infections (right) such as CF lung infections, *P. aeruginosa* growing as biofilms is typically non-motile, is more likely to harbour loss of function *lasR* variants and be protease-deficient. While *P. aeruginosa* may be attenuated in its production of acute virulence factors, it still favours a pro-inflammatory milieu due to a lack of proteolytic degradation of host cytokines and mediators by LasR-regulated proteases. This pro-inflammatory milieu leads to an exuberant neutrophilic response that is largely ineffective at clearing *P. aeruginosa* during chronic infections, but causes collateral tissue damage and lung pathology.

In contrast, *P. aeruginosa* in chronic CF infections grows as biofilm aggregates with cells encased and immobilized in an extracellular matrix, and undergoes extensive pathoadaptation to the CF lung environment. CF-adapted *P. aeruginosa* strains are often non-motile, induce less cytotoxicity and frequently carry loss of function *lasR* variant alleles. Although LasR loss of function might dampen the direct tissue damage inflicted by LasR-regulated factors, our work indicates that protease-deficient *lasR* variants induce an exaggerated neutrophilic inflammation. Using a murine model of subacute lung infection with *P. aeruginosa* embedded in agar beads to approximate CF airway infections, we show that *lasR* variants induce higher levels of pro-inflammatory cytokines, increased airway epithelial mICAM-1 expression as well as greater neutrophilic inflammation and lung pathology than wild-type infections. These results support our *in vitro* findings that the loss of secreted proteases in LasR-deficient variants induce a highly pro-inflammatory milieu, with accumulation of cytokines (e.g. IL-8, IL-6) and mICAM-1, leading to increased recruitment, transmigration and adhesion of neutrophils to the airway epithelium. We thus propose that in chronic infections such as those seen in CF airway infections, the loss of LasR-dependent secreted proteases fuels the exuberant and tissue-damaging neutrophilic inflammation.

LasR quorum sensing and LasR-regulated proteases are involved in complex interactions with host targets, with divergent implications on the pathogenesis of acute and chronic infections. While inhibitors of LasR and secreted proteases have been proposed as anti-virulence therapy for *P. aeruginosa* infections, these therapies should be considered with caution, as they may further exacerbate inflammation and thus worsen the outcomes of chronic *P. aeruginosa* lung infections where excess neutrophils are major drivers of the disease pathology.

